# Supplementary material for: Field-induced bound-state condensation and spin-nematic phase in SrCu2(BO3)2 revealed by neutron scattering up to 25.9 T
Source: Nat Commun. 2024 Jan 10;15:442. doi: 10.1038/s41467-023-44115-z (PMC10781965; doi:10.1038/s41467-023-44115-z)
Supplement: Supplementary file 1 — Supplementary Information [file 41467_2023_44115_MOESM1_ESM.pdf]

Supplementary Information to accompany the article

Field-induced bound-state condensation and spin-nematic phase in $SrCu_2(BO_3)_2$ revealed by neutron scattering up to 25.9 T

Ellen Fogh, Mithilesh Nayak, Oleksandr Prokhnenko, Maciej Bartkowiak, Koji Munakata, Jian-Rui Soh, Alexandra A. Turrini, Mohamed E. Zayed, Ekaterina Pomjakushina, Hiroshi Kageyama, Hiroyuki Nojiri, Kazuhisa Kakurai, Bruce Normand, Frédéric Mila and Henrik. M. Rønnow

S1. INELASTIC NEUTRON SCATTERING EXPERIMENT

A. Geometric considerations in real and reciprocal space

The HFM/EXED instrument that was built at the HZB is described in a series of detailed publications[1–4]. The sample orientation for our experiment was established by using EXED in diffraction mode with a wavelength range of $\lambda = [0.6, 6.3]$ Å. Three axes are used to define this orientation, $B\overline{u}$, $B\overline{v}$ and $B\overline{u} \times B\overline{v}$, as represented in Fig. S1a. The sample rotation angles with respect to the three axes, $(6.0°, 0.8°, -2.7°)$, were determined by indexing a number of nuclear Bragg peaks and following their position on the detector at different magnet rotation angles, φ. The scattering geometry, defined in Fig. S1b, was constrained by the cone-shaped 30° openings of the magnet. The predicted detector image, shown in Fig. S1c, agreed well with the measured image shown in Fig. S1d; these images were collected using $\varphi = -8°$, where five nuclear Bragg peaks were observed. The INS experiment was then carried out with $\varphi = -10°$, where only three Bragg peaks were visible, but the larger rotation angle allowed larger scattering angles, which enlarged the accessible portion of reciprocal space.

The range of $\mathbf{Q}$ accessible on EXED was restricted by the high-field magnet, as Fig. S2a shows for an energy transfer of 3.0 meV, where almost one Brillouin zone is covered. This area was larger at lower energy transfers, but smaller at higher ones. The comparison in Fig. S2a makes clear that on EXED we could not access the high-intensity point at $\mathbf{Q} = (-0.5, 1.5, 0)$, or any of its equivalent points, found in a previous experiment at zero field[5]. While the structure factors at finite fields are not known, the earlier zero-field data showed that different excitations have markedly different structure factors, but also that this difference is detectable only at higher absolute values of $\mathbf{Q}$. As a result, the only $\mathbf{Q}$-dependence we could observe on EXED was an overall increase of intensity with increasing $|\mathbf{Q}|$.

The range accessible along $(0, 0, Q_l)$ was restricted by the choice of Q_h and Q_k as well as by the energy transfer, as shown in Fig. S2b. Thus a given point in the spectrum represents a certain range of Q_l values and consequently a specific ratio of the measured dynamical structure factors, because $S^{zz} \propto (1 - \hat{Q}_l^2)$ whereas $S^{xx} \propto \frac{1}{2}(1 + \hat{Q}_l^2)$; these two quantities are illustrated in Figs. S2c-d. For the $\mathbf{Q}$-integrated spectra shown in Figs. 2g-l of the main text, higher energies contain stronger contributions from $S^{xx}(\mathbf{Q}, \omega)$ whereas lower energies are more reflective of $S^{zz}(\mathbf{Q}, \omega)$. We comment that $Q_k = 0$ was used when calculating $\hat{Q}_l^2$ in Fig. S2c-d, although in practice the integration range along $(0, Q_k, 0)$ was rather large, as in Figs. 2g-l of the main text.

B. Data treatment and background subtraction

The INS data were corrected for the detector efficiency following a standard procedure where individual detector elements are normalized with respect to a vanadium standard. This correction and all subsequent cuts through the data were performed using the Mantid software package[6]. Figures 2g-l of the main text, and the slices of Fig. 1e at the same fields, show the background-subtracted neutron intensity integrated over the entire available area of reciprocal space, $Q_h = [-0.25, 1.25]$, $Q_k = [-0.75, 0.75]$ and $Q_l = [-0.9, 1.3]$, for the data collected with $E_i = 8$ meV. This background was based on the zero-field data, where it is known that the lowest-lying excitation is the one-triplon mode at 3.0 meV[5,7–10], and at the low temperature of our experiment one expects a signal only on the neutron energy-loss side of the spectrum. The background was defined by a three-step fitting procedure that we illustrate in Fig. S3. (i) Quasi-elastic scattering was approximated by a Lorentzian. (ii) After subtracting the Lorentzian, the elastic lineshape was fitted to six Gaussians. This number is purely empirical and was optimized to describe the shape of the elastic line. (iii) Once both Lorentzian and Gaussian contributions had been subtracted, the time-independent part of the background was fitted to the form $I(E) = I_0(E_i - E)^{-3/2}$, with $E_i = 8$ meV fixed and the constant I_0 as the only fitting parameter. The final background is then a sum of the three contributions.

For the intensity data measured with $E_i = 4$ meV and shown in Fig. 3a of the main text, the integration range was $Q_h = [-0.15, 0.85]$, $Q_k = [-0.5, 0.5]$ and $Q_l = [-1.5, 2.5]$. The zero-field results in this case did not have good statistics because the counting time was only 10 minutes, and instead we based our background estimate on the 25.9 T data, which were collected for 5.5 h. Because the spectrum is not known at this field, the background estimation procedure was based on data points in the energy interval $E = [-4.0, 0.1]$ meV.

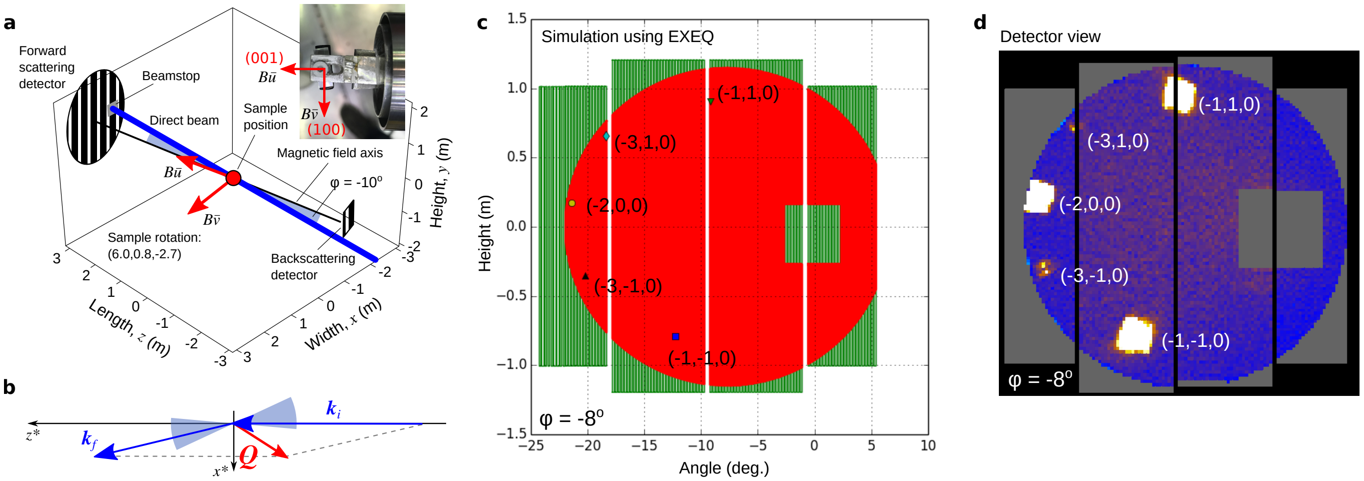

FIG. S1. **Experimental setup. a** Schematic representation of the instrument geometry, showing the magnet axis (black line), the neutron beam (thick blue line) and the detector positions. The axes $B\overline{u}$ and $B\overline{v}$ (red arrows) define the sample orientation. The inset shows a photo of the sample mounted in the dilution refrigerator, indicating the orientation. **b** Scattering triangle showing the wavevectors $\mathbf{k}_i$ of the incoming neutrons, $\mathbf{k}_f$ of the outgoing neutrons and the scattering vector, $\mathbf{Q} = (Q_h, Q_k, Q_l)$. The cones represent the opening angles of the magnet, whose rotation with respect to the incoming beam is φ. **c** Simulated detector view for $\varphi = -8°$, showing the predicted positions of five different nuclear Bragg peaks. **d** Detector view from the experiment with the same settings as in panel **c**. Panels **a** and **c** were generated using the EXEQ prediction tool[11].

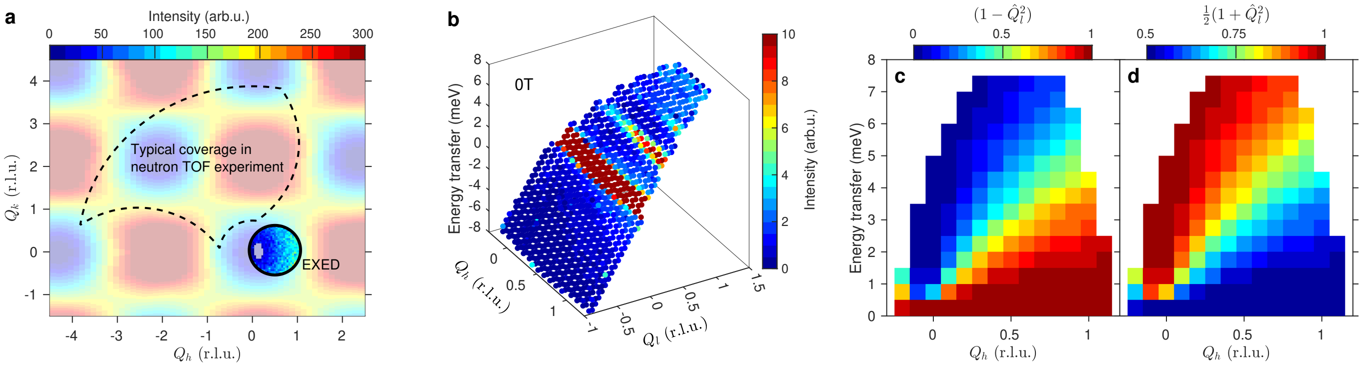

FIG. S2. **Coverage in Q and energy. a** The area inside the solid circle shows the portion of reciprocal space accessible in our EXED experiment and the area inside the dashed lines its comparison with the range covered in a typical time-of-flight INS experiment with $E_i = 8$ meV and detector coverage 10-100°. This slice in $(Q_h, Q_k, 0)$ was made for energies in the range $[2.79, 3.29]$ meV around the one-triplon excitation. The pattern shown with faded colour is the corresponding structure factor calculated from the zero-field MPS spectrum. **b** Representation of the connection between the energy transfer and the Q_l component of the scattering vector. The data shown were taken at zero field and integrated over the range $Q_k = [-0.75, 0.75]$. **c, d** Structure-factor components $(1-\hat{Q}_l^2)$ and $\frac{1}{2}(1+\hat{Q}_l^2)$, shown as functions of energy transfer and of Q_h, again with integration over $Q_k = [-0.75, 0.75]$.

A spurion was observed around 2.1 meV for data with $E_i = 4$ meV and around 5.1 meV for data with $E_i = 8$ meV. This feature was field-independent and appeared close to $\mathbf{Q} = 0$. A Gaussian was fitted and subtracted to remove the spurion before fitting the spectrum of excitations originating in the sample.

C. Peak fitting and intensities

Here we describe in detail the procedure used to fit the data shown in Figs. 2g-l of the main text. Previous theoretical analysis at zero field and our own numerical results at finite fields motivate the presence, positions and widths of a significant number of candidate excitations, as a result of which we based our fit on multiple Gaussian profiles. At zero field, the peak width we obtained for the one-triplon mode around 3.0 meV was used to fix the widths of the excitations at 4.8, 5.5 and 6.5 meV, with the ratio of these widths taken from Ref.[9]. The zero-field parameters of the $S = 1$ two-triplon bound state were used to fix the properties of the $S^z = 0$ branch of this multiplet at finite fields. We did not include Gaussians for the $S^z = \pm 1$ branches of this multiplet, because their

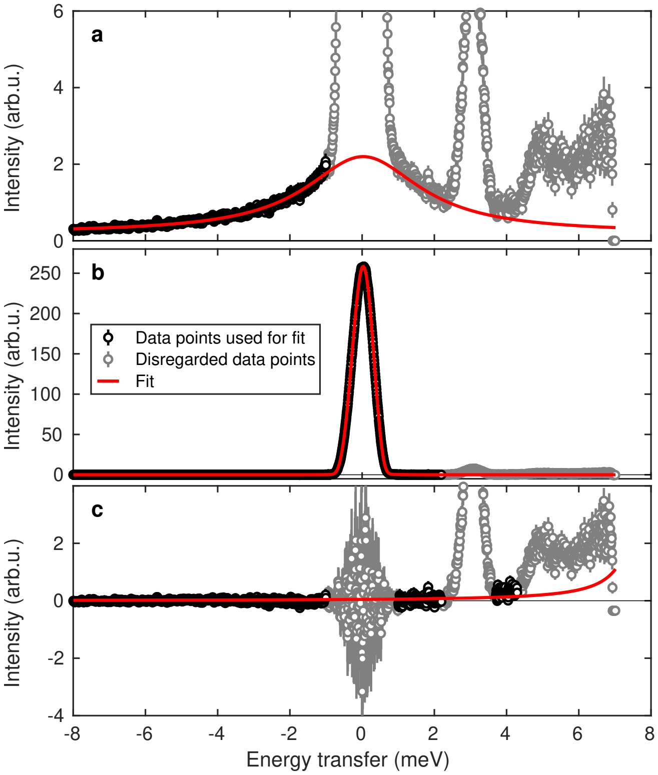

FIG. S3. **Three-step process for background estimation** based on the zero-field data: **a** Lorentzian contribution, **b** elastic line and **c** time-independent part. The black data points are those used for each step of the fitting procedure and the grey ones are those which were disregarded. The red lines show the fit at each step and the final background is their sum. Uncertainties on the neutron counts, N, is $\sqrt{N}$ (Poisson counting statistics) and errors are propagated from the neutron counts taking into account all normalization factors.

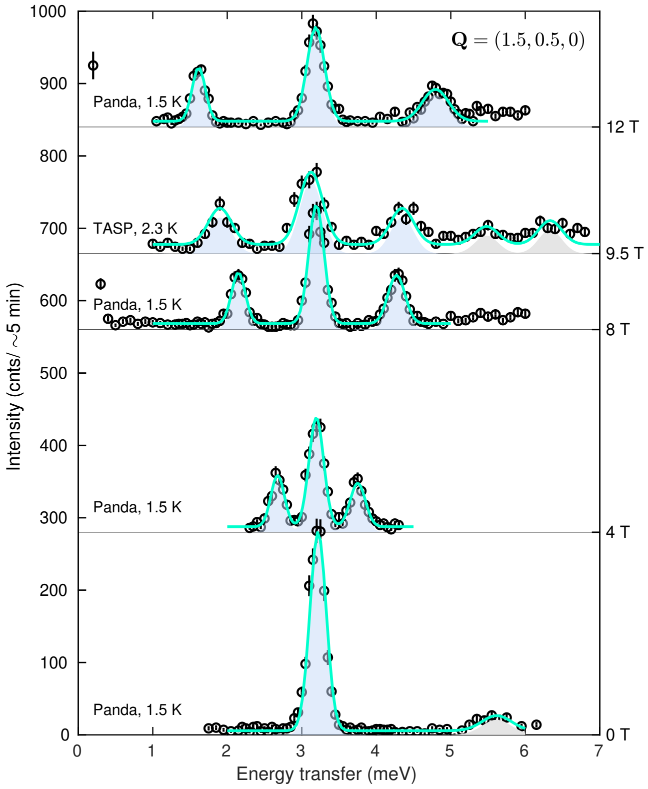

FIG. S4. **Data collected at triple-axis spectrometers below 15 T.** Neutron intensities measured as functions of energy transfer at constant $\mathbf{Q} = (1.5, 0.5, 0)$ on Panda and TASP at selected field strengths. The data points are offset along the vertical axis by a distance proportional to the corresponding applied field value, as noted to the right. The blue shading shows fits to the three one-triplon branches. The grey shading shows additional excitations at higher energies. Uncertainties on the neutron counts, N, is $\sqrt{N}$ (Poisson counting statistics) and errors are propagated from the neutron counts taking into account all normalization factors. The data from TASP were scaled to those from Panda by matching the integrated intensities of the measured t_0 branches. The data collected at 8 T is reprinted with permission from Ref. 10 (Copyright (2023) by the American Physical Society.)

neutron intensities are expected to be weak. We note also that the $S = 1$ bound state has vanishing intensity at $\mathbf{Q} = (1.5, 0.5, 0)$, which is why it cannot be observed in some of the data we show in Fig. 1e of the main text, or here in Fig. S4.

At finite fields, the one-triplon mode undergoes a Zeeman splitting, with the t_- and t_+ branches positioned at energies $\pm\alpha\,\mu_0 H$ from the t_0 branch, where $\alpha \simeq 0.131$ meV/T[12]. We fixed the widths of the t_+, t_- and $S = 1$, $S^z = 0$ bound-state branches to that fitted for the t_0 mode. Only at 15 T, where the t_0 peak is close to the $S_z = +1$ branch of the $S = 1$ multiplet, did we use the fitted width of the t_+ mode, which is well defined at this field, to fix the others. At all measurement fields from 18 to 25.9 T, we found that a single additional Gaussian placed at an energy below the t_0 branch, and up to three additional Gaussians placed below the t_- branch, provided a good fit to the intensity profiles measured as a function of energy transfer. The positions and widths of these Gaussians were allowed to vary freely, except if the widths became much greater or smaller than that of the t_0 branch, in which case their widths were fixed relative to that of this branch. Despite their two-triplon nature, the excitations in this energy and field range are not expected to display a dispersion significantly larger than that due to the DM term[9], and the width of the momentum-integrated t_0 excitation represents this bandwidth. We stress that our fit does not imply the presence of just one additional excitation below the t_0 branch, only that a single Gaussian is suitable for capturing all of the additional intensity arising from these excitations, a quantity we used in Fig. 3d of the main text and analyse in detail in Sec. S2D. For completeness we state that the position of the highest-energy mode below the t_- branch at 23 T was fixed.

In Fig. 1e of the main text, the symbols shown at 0, 15, 18, 20, 23 and 25.9 T were taken from the Gaussians

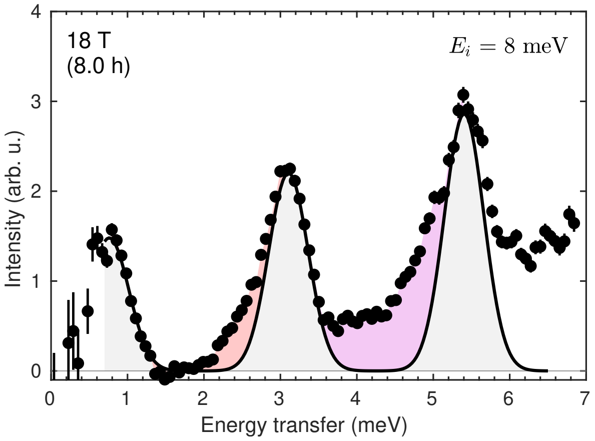

FIG. S5. **Illustration of INS data fitting**, shown for an applied field of 18 T. The one-triplon branches are fitted by three Gaussians of fixed width (black lines and grey shading). Red shading indicates the extra intensity found below the t_0 branch and purple shading the extra intensity appearing below the t_- branch. Uncertainties on the neutron counts, N, is $\sqrt{N}$ (Poisson counting statistics) and errors are propagated from the neutron counts taking into account all normalization factors.

obtained in Figs. 2g-l. The data slices shown at fields between 0 and 15 T were taken from separate experiments: data at 4, 8 and 12 T were measured on Panda, as noted in the Methods section, at 1.5 K and at constant $\mathbf{Q} = (1.5, 0.5, 0)$[10,13]; the data at 9.5 T were measured on TASP, also at constant $\mathbf{Q} = (1.5, 0.5, 0)$. The fitting procedure for these data, which are represented with field-scaled separation in Fig. S4, followed a simplified version of the procedure applied at high fields. For the Panda data, three Gaussians were fitted to the three one-triplon branches, with all parameters allowed to vary. For the TASP data, the three one-triplon branches were fitted with three Gaussians of the same width, with the positions of the t_- and t_+ branches constrained to have the same separation from the t_0 position and with their intensities constrained to have half the intensity of the t_0 excitation. Two additional Gaussians were fitted at higher energies, with their individual positions, widths and intensities taken as free parameters.

Figure 3a of the main text shows the low-energy parts of three INS spectra collected with $E_i = 4$ meV. The momentum-integration ranges and background fitting are described in Sec. S1B. To estimate the peak positions close to the elastic line at 20 and 25.9 T, we fitted one Gaussian whose width was fixed to the value obtained for the t_0 mode at zero field in a separate experiment on HFM/EXED.

Figure 3d of the main text shows the integrated spectral weights found in energy windows below the t_0 and t_- one-triplon branches. To illustrate the extraction of these weights, in Fig. S5 we show the experimental data at a representative field of 18 T. The one-triplon contributions were fitted by three Gaussians following the above procedure. The excess spectral weight induced below t_0 is then defined as that appearing above the one-triplon contribution between t_+ and t_0, highlighted with red shading in Fig. S5 and it is integrated numerically. The excess weight below t_- is defined in the same way and is shown by the purple shading.

D. Q-dependence

As Figs. 2a-f of the main text make clear, the spin excitations of $SrCu_2(BO_3)_2$ are extremely insensitive to $\mathbf{Q}$, and this was the basis on which we integrated our EXED results over the full reciprocal space for further analysis. Here we nevertheless inspect the differences discernible over the $\mathbf{Q}$ range covered in our measurements (Fig. S2a). The top two rows of Fig. S6 (colour panels) represent the intensities measured in the $(Q_h, Q_k, 0)$ plane in energy windows selected to cover the regions below (top row) and spanning (second row) the t_0 excitation branch for all six measurement fields. While the shape of the intensity distribution is indeed very similar in all panels, with the highest intensities at the largest $|Q|$ values, the net intensity reflects the general shift from the t_0 branch to the novel multi-triplon excitations below it (red Gaussians in Figs. 2g-l) as the field is increased, as quantified in Fig. 3d of the main text.

The third row of panels in Fig. S6 quantifies the evolution of the two intensity contributions with Q_h by integrating over $Q_k = [-0.1, 0.1]$ (black boxes in second row). At 0 and 15 T, where no statistically significant extra intensity could be discerned below the t_0 branch (Figs. 2g and 2h of the main text), the intensity in the selected integration window can be attributed to the sample environment; because different energy intervals correspond to different intervals in Q_l, we do not subtract this background contribution. The intensities measured for the t_0 branch at each field show a largely linear dependence on Q_h and a steady decrease as the field is increased above 18 T; the intensities of the multi-triplon contribution below t_0 also increase with Q_h and increase with field until they match the t_0 weight. The bottom row of panels in Fig. S6 shows how the position of the t_0 branch, and the centre position of the spectral weight below t_0, vary weakly with Q_h. The data were discretized in units of $\Delta Q_h = 0.1$ for fields up to 20 T and $\Delta Q_h = 0.2$ at 23 and 25.9 T. The very minor dispersion observed in all cases is consistent with the bandwidth identified in zero-field studies[8,9].

S2. NUMERICAL CALCULATIONS AND ANALYSIS

A. Dependence on cylinder size

An MPS Ansatz describes a one-dimensional (1D) quantum many-body wavefunction. A 2D system can

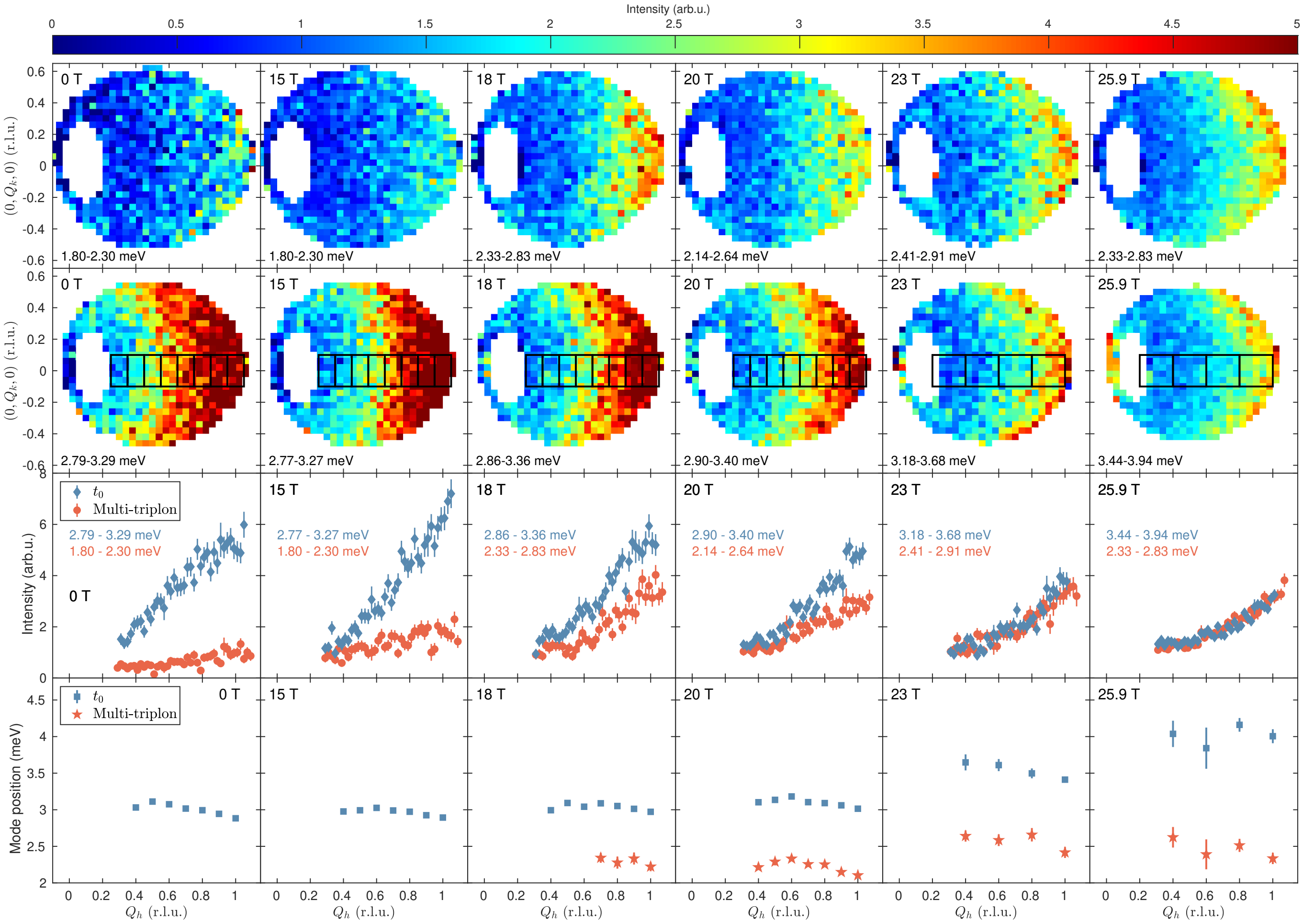

FIG. S6. **Q-dependence of triplet and multi-triplet excitations.** Data slices showing the **Q**-dependence of the neutron intensity below the t_0 excitation branch (top row) and around the t_0 branch (second row). In preparing these slices, the intensity data were integrated over the momentum interval $Q_l = [-1.5, 2.5]$ and over an energy interval of width $\Delta E = 0.5$ meV centred on the fitted positions of the red Gaussians (to obtain the top row) and the t_0 Gaussians (to obtain the second row) in Figs. 2g-l of the main text. The zero-field intensity of the t_0 branch has been divided by 2 for illustration on the same colour scale. Below 18 T, where no extra intensity was detected below t_0, the top two colour panels show simply an energy window well below the t_0 window. The black boxes in the second row illustrate the region of integration used to produce the panels in the third and fourth rows. These show respectively the integrated intensities and mode positions as a function of Q_h. In the third row, uncertainties on the neutron counts, N, is $\sqrt{N}$ (Poisson counting statistics) and errors are propagated from the neutron counts taking into account all normalization factors. In the fourth row, the error bars represent the standard deviations given by the least-squares fitting of the mode positions.

be reformulated as a 1D model on a cylinder with long-ranged interactions[14], such that MPS-based DMRG[15,16] can be used to find the ground state and compute the associated static observables. The dynamical structure factor (DSF) of a 2D system can also be computed using MPS-based methods, and for this work we applied the TDVP[17–19], as described in the Methods section. Here we calibrate how the finite length and circumference of the cylinder affects our calculations. All results reported in the main text were obtained using a cylinder circumference of $W = 4$ sites and a length of $L = 20$ sites, as represented in Fig. S7a. As a first step towards justifying that these values of W and L return results fully representative of the 2D SSM, we computed the ground-state energy and field-induced static magnetization with $W = 6$ (Fig. S7b), finding that both quantities change rather little.

TDVP calculations become extremely time-intensive on wider cylinders for two reasons. First the bond dimension of the MPS should increase exponentially with W in order to describe the state with equivalent accuracy. Second, wider cylinders introduce further-neighbour interactions in the effective 1D model that increase the virtual dimension of the Matrix Product Operator in proportion with W. The resulting $O(W^2 \exp(3W))$ scaling of the calculation time therefore prevented us from obtaining the DSF with $W = 6$ at all values of the magnetic field required for our study, but we did compute it at 15

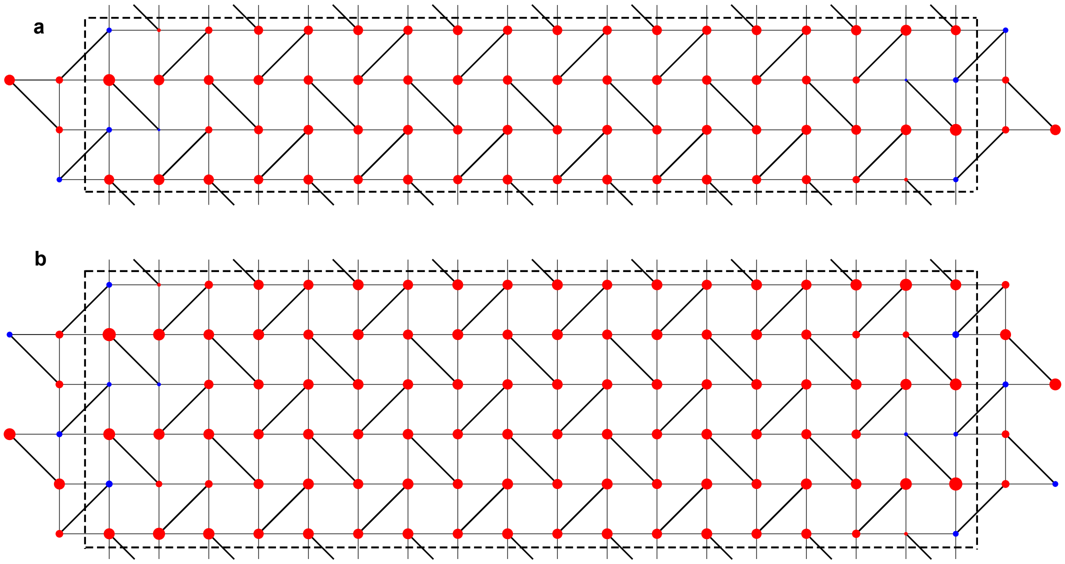

FIG. S7. **Illustration of the cylinder geometries** used for our MPS calculations. **a** Cylinder width $W = 4$. **b** $W = 6$. The red (positive) and blue (negative) circles indicate the magnetization per site obtained at $H = 20$ T, and the degree to which it is uniform across the central regions of the cylinder. The dashed rectangle indicates the region used for the spatial Fourier transform.

and 20 T, and in Fig. S8 we compare the **Q**-integrated DSFs obtained for $W = 4$ and 6 at both fields. Because the allowed k_y values for a $W = 4$ cylinder are 0 and π, while for a $W = 6$ cylinder they are 0, $2\pi/3$ and $4\pi/3$, increasing W results in more spectral weight for each mode and thus we normalize the spectra to the same integrated weight. At first sight there is remarkably little change from $W = 4$ to 6, and on close inspection one may ascertain that the loss of one-triplon peak height in the $W = 6$ spectrum reappears at energies between and above the three primary branches. This could be expected on the grounds that the wider cylinder allows more space for the development of multi-triplon dynamics. Overall, however, the effects of W are small and the effects of L are negligible beyond $L = 20$; while we do not show the latter null result, it is readily understood from the extreme localization of all one- and higher-triplon excitations in the ideally frustrated SSM geometry.

B. Role of Dzyaloshinskii-Moriya interactions

As discussed in the main text, a primary consequence of two-triplon bound-state condensation is the presence of a one-triplon gap at all fields. However, in $SrCu_2(BO_3)_2$ there are weak DM interactions on both the intra- and inter-dimer bonds, as represented in Fig. 1b of the main text, and in particular the intra-dimer D term leads to a direct mixing of S^z sectors in the SSM. It is known from studies of a spin ladder with intra-dimer DM terms that an avoided crossing can occur, such that the one-triplon gap reaches a finite minimum at the expected condensation field, and then increases again at

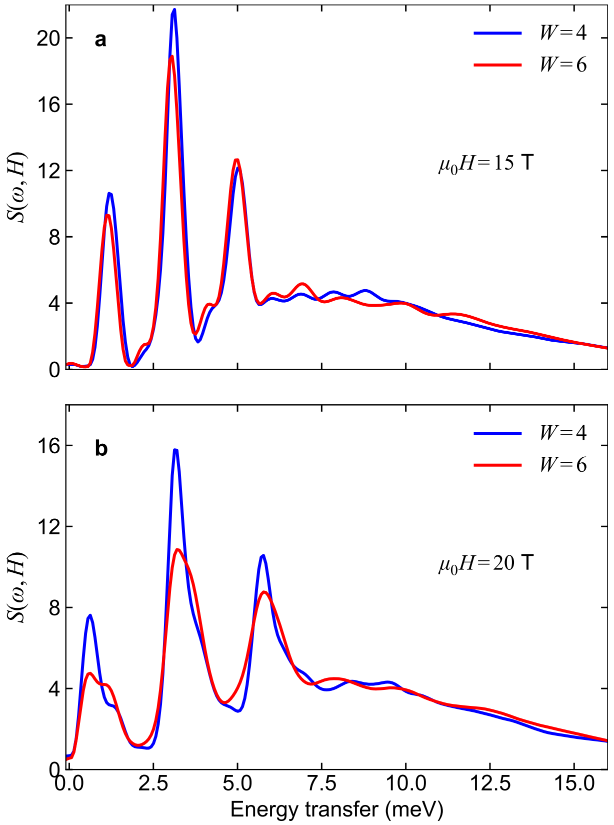

FIG. S8. **Effect of cylinder size.** Comparison of fully **Q**-integrated dynamical structure factors, shown as a function of energy transfer, computed using cylinders of widths $W = 4$ and 6 at 15 T (**a**) and 20 T (**b**).

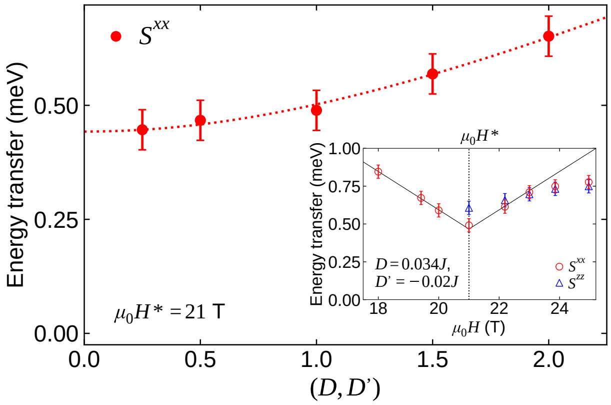

FIG. S9. **Gap size due to DM interactions.** Excitation energies obtained by MPS calculations on cylinders of width $W = 4$ to illustrate the effect of the DM interactions at a field equivalent to $\mu_0 H^* = 21$ T. On the x-axis, D and D' are scaled to their experimental values. On the y-axis, the position of the t_+ branch was obtained from the lowest peak in the S^{xx} component of the dynamical susceptibility and the error bar indicates the width of this peak. Variation of the t_+ gap with magnetic field at fixed $D = 0.034J$ and $D' = -0.02J$ is shown in the inset. For comparison we show the peak energy of the low-energy spectral weight in the S^{zz} component, which arises from internal excitations within the $|t_+ t_+\rangle$ bands (Fig. 4a of the main text). The black solid line corresponds to the behavior of the one-triplon branch as predicted for bound-state condensation without DM interactions (Fig. 1d of the main text).

higher fields[20]. To eliminate the possibility that the behaviour we observe is a DM-induced avoided crossing, we have performed MPS calculations at different values of D to identify the evolution of the lowest-lying excitations. We note again that a small but finite D is required to obtain a uniformly magnetized ground state in our calculations, and for all the MPS results shown in the main text this was set to the experimentally deduced values $D = 0.034J$[21], with $D' = -0.02J$[22]. MPS results for the $D, D' = 0$ limit in the presence of a field may be obtained by varying both parameters and extrapolating the results.

We computed the transverse (S^{xx}) and longitudinal (S^{zz}) components of the dynamical susceptibility at $\mu_0 H = 21$ T for four different D values, with D' fixed to $-0.59D$, and extracted the lowest-lying excitations in each sector. The one-triplon gap, read from S^{xx} by fitting a Gaussian to the low-energy spectral weight, is shown in Fig. S9. For comparison we show the generic functional form $\Delta(D) = \sqrt{\Delta_0^2 + c^2 D^2}$, which interpolates between a constant at $D = 0$ and a linear increase at large D. This simple form offers a convincing fit, demonstrating that the lower bound for the one-triplon gap as $D \to 0$ is $\Delta_0 = 0.45$ meV. In the inset of Fig. S9, we show that this gap is also not a consequence of the field choice, as varying H confirms the linear increase away from $\mu_0 H^* = 21$ T with both increasing and decreasing field, as represented qualitatively in Fig. 1e of the main text. For completeness we show also the peak energy determined in the same way from S^{zz}, which appears as a result of internal excitations of the two-triplon bound state (Figs. 4a and 4f of the main text). If one accepts the result of Sec. S2A, that this constant minimum gap of order 0.5 meV at H^* is not a finite-size effect, it must therefore be an intrinsic property of the pure SSM (i.e. of the model with only Heisenberg interactions). We repeat the analogy to superconductivity drawn in the main text, where a single-particle excitation formed by pair-breaking in the condensate has an energy gap equal to half the binding energy. This implies that the two-triplon binding energy in $SrCu_2(BO_3)_2$ is around 1 meV, a value fully consistent with the results of third-order perturbation theory[23].

C. Q-dependence of MPS results

A full comparison of our cylinder MPS results with the DSF measured in experiment requires the inclusion of the structure factor of $SrCu_2(BO_3)_2$, as noted in the Methods section. When the DSF is integrated over a large volume in reciprocal space, as in Figs. 1e, 1f, 2, 4a and 4b of the main text, the effect of the structure factor is largely invisible, and for this reason we do not perform extensive comparisons of the **Q**-dependence in our INS and MPS results. To illustrate the role of the structure factor, for example in preparing constant-energy slices of the type shown in Fig. S6, we integrate the DSF obtained from MPS over energy windows selected to highlight a particular excitation feature and display the results in the space of (Q_h, Q_k). Our MPS calculations allow the further advantage of separating the excitations overlapping in a specific energy window by considering the transverse and longitudinal channels separately. In Fig. S10 we show a number of constant-energy slices prepared from our MPS data at a field equivalent to 25 T. The t_0 branch of the one-triplon excitation is found in the longitudinal (S^{zz}) channel and its **Q**-dependence is shown in Fig. S10a by selecting an energy window of 0.5 meV around the field-induced centre position of 4.05 meV. Similarly, the internal excitations of the $|t_+ t_+\rangle$ pinwheel, shown in Fig. 4a of the main text, are found at low energies in S^{zz} (Fig. S10b). Otherwise, stronger spectral contributions from the novel multi-triplon excitations appear in the transverse (S^{xx}) channel, and in Figs. S10c-e we compare the intensity distributions for energy windows below the t_0 branch (Fig. S10c), around the t_0 branch but in the opposite channel (Fig. S10d) and below the t_- branch (Fig. S10e). In each case we observe distinctive patterns that could be analysed in future high-field INS experiments.

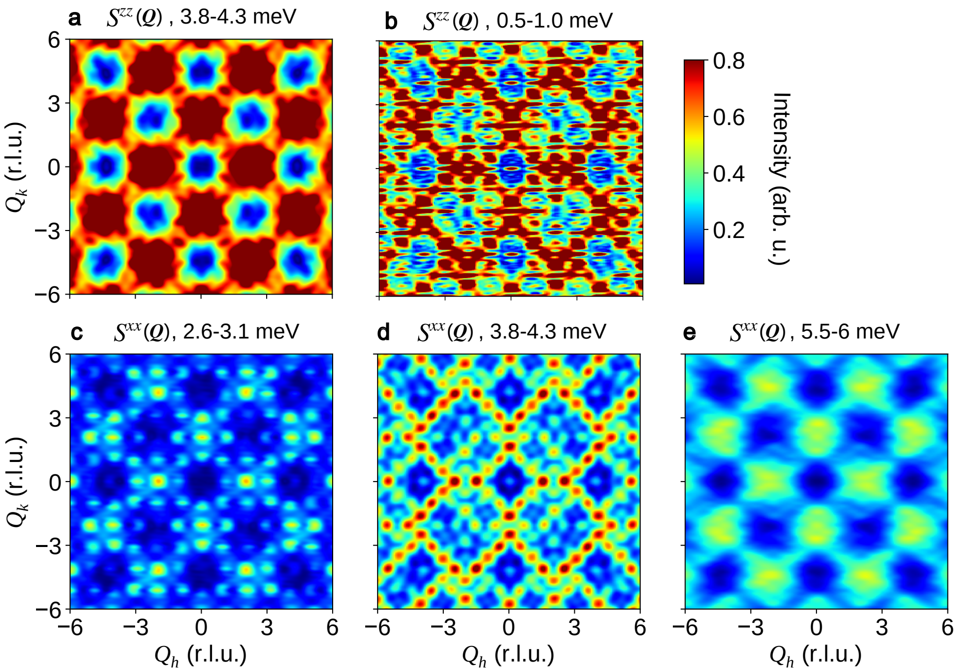

FIG. S10. **Constant-energy slices at 25 T** displayed in (Q_h, Q_k). **a** Slice including the one-triplon t^0 branch extracted from S^{zz}. **b** Slice including the internal excitations of the two-triplon bound state, extracted from S^{zz}. **c**-**e** Slices including different multi-triplon bound states extracted from S^{xx} for energy intervals 2.6-3.1 (**c**), 3.8-4.3 (**d**) and 5.5-6.0 meV (**e**). We comment that the weak breaking of fourfold symmetry visible in these panels is a consequence of the cylinder geometry (20×4, Fig. S7a).

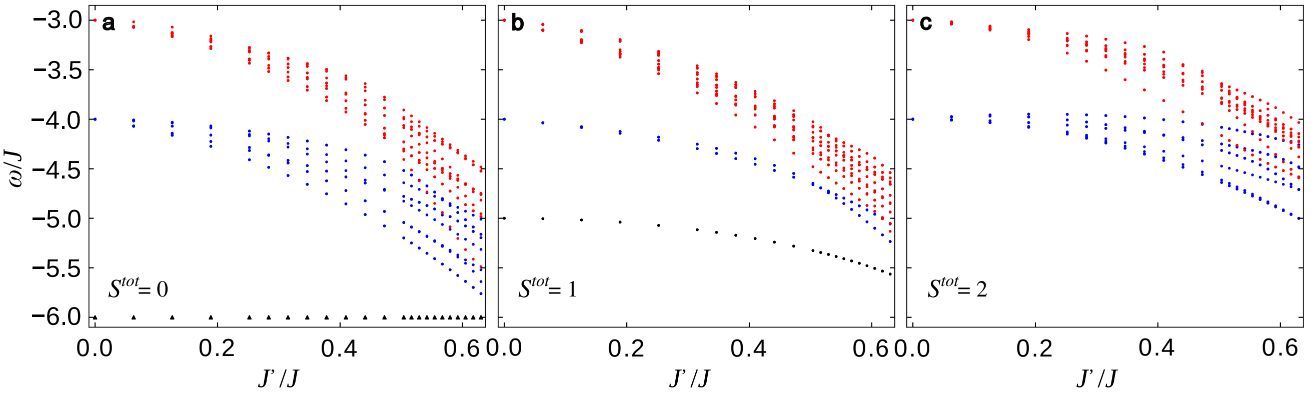

FIG. S11. **Evaluation of the excitation spectrum.** Energy levels of the SSM obtained by ED of a 4×4 spin cluster. Results are shown as a function of J'/J at zero field for the separate total-spin (**a** $S^{\rm tot} = 0$, **b** $S^{\rm tot} = 1$ and **c** $S^{\rm tot} = 2$) sectors at momentum $(k_x, k_y) = (0, 0)$. Black triangles mark the singlet ground state (panel **a**), black circles the three one-triplon states (panel **b**), blue circles the two-triplon states and red circles the three-triplon states (all panels).

D. Identification of multi-triplet excitations

To identify the nature of the excited states found in our cylinder MPS calculations (Figs. 1f and 4a-b of the main text), we exploit the fact that the DM interactions of the system are too weak to introduce confusion (by a mixing of spin sectors) and neglect them completely. For identification purposes it is fully sufficient to use a pure SSM and to perform ED[24] on a cluster of only 4×4 spins, where (as noted above) the available k_x and k_y momentum sectors are 0 and π. The ED spectrum is computed in symmetry sectors labelled by the quantum numbers of total spin ($S^{\rm tot}$), total magnetization (S^z) and the momenta k_x and k_y, and we make use of the separate S^{zz} and S^{xx} components of the DSF to identify states from the action of the S^z and S^x spin operators.

As discussed in the main text, the two-triplon bound state, $|t_+t_+\rangle$, is the first to condense as the applied magnetic field is increased. This state belongs to the symmetry sector $S = 2$, $S^z = 2$, and its lowest-energy component appears at $(k_x, k_y) = (0, 0)$ on the 4×4 cluster. In the limit $J'/J \to 0$, the energies of n-triplon states

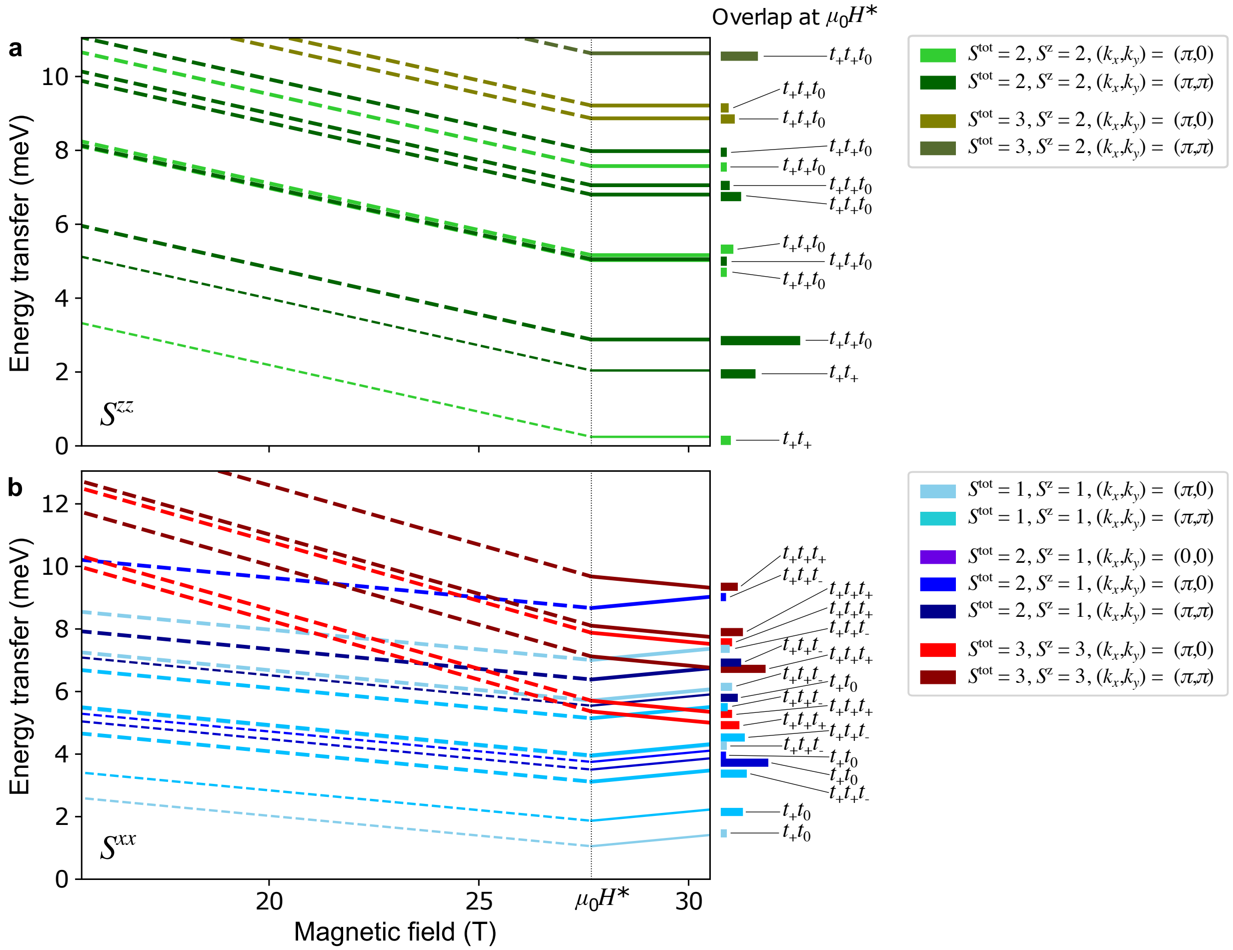

FIG. S12. **Identification of composite multi-triplon states** in the spectrum of the 4×4 SSM spin cluster in a magnetic field strong enough to condense the lowest $|t_+t_+\rangle$ state. **a** Excitations accessible in the longitudinal component ($S^{zz}(k,\omega,H)$) of the DSF. **b** Excitations accessible in the transverse component ($S^{xx}(k,\omega,H)$) of the DSF. On this cluster, the lowest-energy component of the two-triplon bound state reaches the singlet energy at a field equivalent to $\mu_0 H^* = 27.7$ T when using the g-factor of $SrCu_2(BO_3)_2$. We show all of the higher-weight states appearing at low energies in the momentum sectors $(0,0)$, $(\pi,0)$ and (π,π). Because the spin Hamiltonian is of pure Heisenberg type, the field term causes only a linear evolution whose gradient is determined by S^z, with a kink and a $\Delta S^z = 2$ change of slope at H^*.

approach nJ, and thus all the states in the spectrum are readily identified by following the evolution of their energies with J'/J, as we show in Fig. S11 for the lowest three $S^{\rm tot}$ sectors. It is clear that the different n-triplon sectors remain well separated in energy for smaller values of $J'/J = 0$, but that their energies show significant overlap as the interaction ratio approaches the $SrCu_2(BO_3)_2$ value of $J'/J = 0.63$.

For more detailed insight into the character of each n-triplon sector, we consider the action of the operators S^z and S^x on the two-triplon bound state. From the Lehmann representation of the DSF, the excitations of a given initial state lie in symmetry sectors allowed by the selection rules

1. $S^{zz}(k_x,k_y)$: $\Delta S = 0,1$; $\Delta S^z = 0$; $k_x = k_{x,f} - k_{x,i}$; $k_y = k_{y,f} - k_{y,i}$;
2. $S^{xx}(k_x,k_y)$: $\Delta S = \pm 1$; $\Delta S^z = \pm 1$; $k_x = k_{x,f} - k_{x,i}$; $k_y = k_{y,f} - k_{y,i}$.

The excitations allowed by these rules do not contribute equally to the spectral weight, because all have different matrix elements. To deduce the contribution of each state in the spectrum, we compute the overlap

$$\mathcal{N} = \left|\left\langle \psi_{\rm e} | S^{\alpha}_{k_x,k_y} | \psi_0 \right\rangle\right|^2, \qquad (1)$$

where $\alpha = x, z$, $|\psi_0\rangle$ is the ground (initial) state and $|\psi_{\rm e}\rangle$ is the excited (final) state. We take $\mathcal{N} \geq 0.01$ as the criterion to regard $|\psi_{\rm e}\rangle$ as a state making a significant contribution to the DSF. We note that, while the initial state is always the lowest-energy state of the $(S, S^z, k_x, k_y) = (2, 2, 0, 0)$ sector, this is not in fact the ground state at $H < H^*$.

The final step is to deduce the energies of these high-weight levels in the presence of a magnetic field. Because

of the purely Heisenberg nature of the Hamiltonian, every state undergoes a simple Zeeman shift determined by its S^z quantum number, giving

$$\omega_{\mathrm{e}}(H) = E_{\mathrm{e}} - E_0 + (S_0^z - S_{\mathrm{e}}^z)H, \qquad (2)$$

which allows us to track the origin of all the observed states at low fields, as we show in Fig. S12. The small cluster size we have used for this exercise results in non-trivial finite-size effects on the energy levels: in a field, the t_+ branch crosses the singlet before the lowest $|t_+t_+\rangle$ branch, and the crossing field defined from the latter is $\mu_0 H = 27.7$ T. However, these quantitative details are not relevant for the qualitative purpose of identifying the high-weight excitations appearing in an INS experiment on a system with a two-triplon bound-state condensate (i.e. a spin nematic) as the field-induced ground state. Figure S12 provides the full justification for the statements made in the main text that the many excitations visible in the INS and MPS spectral functions are composite two- and three-triplon excitations of several different types.

[1] O. Prokhnenko, W.-D. Stein, H.-J. Bleif, M. Fromme, M. Bartkowiak, and T. Wilpert, "Time-of-flight Extreme Environment Diffractometer at the Helmholtz-Zentrum Berlin," Rev. Sci. Instrum. **86**, 033102 (2015).

[2] P. Smeibidl, M. Bird, H. Ehmler, I. Dixon, J. Heinrich, M. Hoffmann, S. Kempfer, S. Bole, J. Toth, O. Prokhnenko, and B. Lake, "First Hybrid Magnet for Neutron Scattering at Helmholtz-Zentrum Berlin," IEEE Trans. Appl. Supercond. **26**, 4301606 (2016).

[3] O. Prokhnenko, M. Bartkowiak, W.-D. Stein, N. Stuesser, H.-J. Bleif, M. Fromme, K. Prokes, P. Smeibidl, M. Bird, and B. Lake, "HFM-EXED - the high field facility for neutron scattering at HZB," Proceedings of ICANS-XXI , 278–285 (2016).

[4] O. Prokhnenko, P. Smeibidl, W. D. Stein, M. Bartkowiak, and N. Stuesser, "HFM/EXED: The High Magnetic Field Facility for Neutron Scattering at BER II," JLSRF **3**, A115 (2017).

[5] D. Lançon, *Neutron spectroscopy in the layered quantum magnet $SrCu_2(BO_3)_2$ and in transition metal phosphorus trisulfides (MPS_3)*, Ph.D. thesis, Ecole Polytechnique Fédérale de Lausanne (2017).

[6] O. Arnold, J. C. Bilheux, J. M. Borreguero, A. Buts, S. I. Campbell, L. Chapon, M. Doucet, N. Draper, R. Ferraz Leal, M. A. Gigg, V. E. Lynch, A. Markvardsen, D. J. Mikkelson, R. L. Mikkelson, R. Miller, K. Palmen, P. Parker, G. Passos, T. G. Perring, P. F. Peterson, S. Ren, M. A. Reuter, A. T. Savici, J. W. Taylor, R. J. Taylor, R. Tolchenov, W. Zhou, and J. Zikovsky, "Mantid - Data analysis and visualization package for neutron scattering and μSR experiments," Nucl. Instrum. Methods Phys. Res. A **764**, 156–166 (2014).

[7] H. Kageyama, M. Nishi, N. Aso, K. Onizuka, T. Yosihama, K. Nukui, K. Kodama, K. Kakurai, and Y. Ueda, "Direct evidence for the localized single-triplet excitations and the dispersive multitriplet excitations in $SrCu_2(BO_3)_2$," Phys. Rev. Lett. **84**, 5876 (2000).

[8] B. D. Gaulin, S. H. Lee, S. Haravifard, J. P. Castellan, A. J. Berlinsky, H. A. Dabkowska, Y. Qiu, and J. R. D. Copley, "High-Resolution Study of Spin Excitations in the Singlet Ground State of $SrCu_2(BO_3)_2$," Phys. Rev. Lett. **93**, 267202 (2004).

[9] K. Kakurai, K. Nukui, N. Aso, M. Nishi, H. Kadowaki, H. Kageyama, Y. Ueda, L-P. Regnault, and O. Cépas, "Neutron Scattering Investigation on Quantum Spin System $SrCu_2(BO_3)_2$," Prog. Theor. Phys. Suppl. **159**, 22 (2005).

[10] M. E. Zayed, Ch. Rüegg, Th. Strässle, U. Stuhr, B. Roessli, M. Ay, J. Mesot, P. Link, E. Pomjakushina, M. Stingaciu, K. Conder, and H. M. Rønnow, "Correlated decay of triplet excitations in the Shastry-Sutherland compound $SrCu_2(BO_3)_2$," Phys. Rev. Lett. **113**, 067201 (2014).

[11] M. Bartkowiak, K. Prokes, M. Fromme, A. Budack, J. Dirlick, and O. Prokhnenko, "EXEQ and InEXEQ: software tools for experiment planning at the Extreme Environment Diffractometer," J. Appl. Cryst **53**, 1613–1619 (2020).

[12] H. Nojiri, H. Kageyama, Y. Ueda, and M. Motokawa, "ESR study on the excited state energy spectrum of $SrCu_2(BO_3)_2$ – a central role of multiple-triplet bound states," J. Phys. Soc. Jpn. **72**, 3243 (2003).

[13] M. Zayed, *Novel States in Magnetic Materials under Extreme Conditions: A High Pressure Neutron Scattering Study of the Shastry-Sutherland compound $SrCu_2(BO_3)_2$*, Ph.D. thesis, Ecole Polytechnique Fédérale de Lausanne (2010).

[14] E. M. Stoudenmire and Steven R. White, "Studying Two-Dimensional Systems with the Density Matrix Renormalization Group," Annu. Rev. Condens. Matter Phys. **3**, 111–128 (2012).

[15] S. R. White, "Density matrix formulation for quantum renormalization groups," Phys. Rev. Lett. **69**, 2863–2866 (1992).

[16] U. Schollwöck, "The density-matrix renormalization group in the age of matrix product states," Ann. Phys. **326**, 96 – 192 (2011).

[17] J. Haegeman, J. I. Cirac, T. J. Osborne, I. Pižorn, H. Verschelde, and F. Verstraete, "Time-dependent variational principle for quantum lattices," Phys. Rev. Lett. **107**, 070601 (2011).

[18] J. Haegeman, Ch. Lubich, I. Oseledets, B. Vandereycken, and F. Verstraete, "Unifying time evolution and optimization with matrix product states," Phys. Rev. B **94**, 165116 (2016).

[19] S. Paeckel, T. Köhler, A. Swoboda, S. R. Manmana, U. Schollwöck, and C. Hubig, "Time-evolution methods for matrix-product states," Ann. Phys. **411**, 167998 (2019).

[20] S. Miyahara, J.-B. Fouet, S. R. Manmana, R. M. Noack, H. Mayaffre, I. Sheikin, C. Berthier, and F. Mila, "Uniform and staggered magnetizations induced by Dzyaloshinskii-Moriya interactions in isolated and coupled spin-1/2 dimers in a magnetic field," Phys. Rev. B **75**, 184402 (2007).

[21] K. Kodama, S. Miyahara, M. Takigawa, M. Horvatić, C. Berthier, F. Mila, H. Kageyama, and Y. Ueda, “Field-induced effects of anisotropic magnetic interactions in $SrCu_2(BO_3)_2$,” J. Phys. Condens. Matter **17**, L61 (2005).

[22] O. Cépas, K. Kakurai, L. P. Regnault, T. Ziman, J. P. Boucher, N. Aso, M. Nishi, H. Kageyama, and Y. Ueda, “Dzyaloshinskii-Moriya Interaction in the 2D Spin Gap System $SrCu_2(BO_3)_2$,” Phys. Rev. Lett. **87**, 167205 (2001).

[23] T. Momoi and K. Totsuka, “Magnetization plateaus of the Shastry-Sutherland model for $SrCu_2(BO_3)_2$: Spin-density wave, supersolid, and bound states,” Phys. Rev. B **62**, 15067–15078 (2000).

[24] A. Sandvik, “Computational Studies of Quantum Spin Systems,” AIP Conf. Proc. **1297**, 135–338 (2010).
